# Segmenting Brain Tissues from Chinese Visible Human Dataset by Deep-Learned Features with Stacked Autoencoder

**DOI:** 10.1155/2016/5284586

**Published:** 2016-01-26

**Authors:** Guangjun Zhao, Xuchu Wang, Yanmin Niu, Liwen Tan, Shao-Xiang Zhang

**Affiliations:** ^1^Key Laboratory of Optoelectronic Technology and Systems of Ministry of Education, College of Optoelectronic Engineering, Chongqing University, Chongqing 400044, China; ^2^College of Computer and Information Science, Chongqing Normal University, Chongqing 400050, China; ^3^Institute of Digital Medicine, College of Biomedical Engineering, Third Military Medical University, Chongqing 400038, China

## Abstract

Cryosection brain images in Chinese Visible Human (CVH) dataset contain rich anatomical structure information of tissues because of its high resolution (e.g., 0.167 mm per pixel). Fast and accurate segmentation of these images into white matter, gray matter, and cerebrospinal fluid plays a critical role in analyzing and measuring the anatomical structures of human brain. However, most existing automated segmentation methods are designed for computed tomography or magnetic resonance imaging data, and they may not be applicable for cryosection images due to the imaging difference. In this paper, we propose a supervised learning-based CVH brain tissues segmentation method that uses stacked autoencoder (SAE) to automatically learn the deep feature representations. Specifically, our model includes two successive parts where two three-layer SAEs take image patches as input to learn the complex anatomical feature representation, and then these features are sent to Softmax classifier for inferring the labels. Experimental results validated the effectiveness of our method and showed that it outperformed four other classical brain tissue detection strategies. Furthermore, we reconstructed three-dimensional surfaces of these tissues, which show their potential in exploring the high-resolution anatomical structures of human brain.

## 1. Introduction

The anatomical structures of the brain tissues are very complex and associated with a number of neurological diseases. Nevertheless, without segmentation, the computer cannot recognize and define a tissue's contour automatically, and the anatomical images are difficult to be used for lateral medical application [1]. Cryosection images in the Chinese Visible Human (CVH) dataset show the true color of the human body in a high spatial resolution and contain more rich and original details of the brain anatomy than other medical imaging, such as CT and MRI [2]. By segmenting CVH brain tissues into cerebrospinal fluid (CSF), gray matter (GM), white matter (WM), or other anatomical structures, we can study human brain and apply it in various fields, such as anatomical education, medical image interpretation, and disease diagnosis [3].

It is known that automatic or semiautomatic segmentation is helpful for alleviating the laborious and time-consuming manual segment; however, much noise is introduced during CVH image acquisition and the image contrast is low at some positions because of the asymmetric illumination. In addition, the CVH dataset has no other similar datasets as atlas for guiding segmentation. So there remains a challenging problem of how to explore new model to segment the whole hundreds of CVH brain images in high accuracy and efficiency.

Currently, most existing brain segmentation algorithms are based on CT or MRI images. According to whether the objects are labeled, these methods can be classified into two categories: unsupervised-based and supervised-based. The unsupervised methods, such as region growing, thresholding, clustering, and statistical models, directly use the image intensity to search the object. For example, the fuzzy *c*-means method classifies image by grouping similar data that are present into clusters and varying the degree of membership function allows the voxel to belong to the multiple classes [4, 5]. This assumption may not work well as it only considers intensity of image and intensity is not enough to express the intrinsic feature of objects. In addition, some methods estimate distribution of each class with probability density of Gaussian mixture model [6]. These methods need accurate estimation of probability density function and for those images with largely overlapped tissues, it is hard to match real distribution of data in a high accuracy. Other methods, like region growing, extend threshold by combining it with connectivity, but they need seeds for each region and have the problem for determining suitable threshold for homogeneity. Also, the gradient-based segmentation technique like Watershed [7] constructs many dams for segmenting image, but it is easy to produce oversegmentation.

Supervised learning-based segmentation methods are promising as they take expert information (labeled data or atlas) into the procedure of segmentation. These methods have shown remarkable improvements in segmenting CT or MRI brain images. For example, Anbeek et al. [7, 8] proposed applying spatial and intensity features in a population-specific atlas space to label brain voxels. This method achieves high accuracy in the cost of a large set of manually segmented training images. The studies in [9, 10] proposed using cooccurrence texture features of wavelet followed by a classifier (like support vector machine) for brain segmentation, while the performance might vary from different dataset at hand with this kind of low-level feature, especially for the cryosection images that contain many of anatomical structures and morphological changes.

When applying supervised methods to segment the obscure targets, there is a common sense that the key to success is mainly dependent on the choice of data representation used to characterize the input data [11]. Typical features, such as histogram [12], texture [13], and wavelet [10, 14], have been successfully applied to many different occasions. But unfortunately, most of these low-level features are hard to extract and organize salient information from the data and their representation power varied from different datasets. Considering that CVH dataset contains hundreds of brain images with enormous anatomical information of different tissues, due to efficiency, we may not label all the images as training data but just a tiny fraction, so it is crucial to extract better feature representation of the inputs so as to infer the labels distribution of the unknown anatomical structures.

Recently, deep neural networks (DNN) have shown their promising results for feature extraction in many computer vision applications [15, 16]. Contrary to traditional shallow classifiers in which feature engineering is crucial, deep learning methods automatically learn hierarchies of relevant features directly from the raw inputs [17]. There are several DNN-based models such as convolutional neural networks (CNNs) [16], deep belief network (DBN) [18], stacked autoencoder (SAE) [19–21] that have been applied in different tasks. As a typical example of deep models, CNNs alternatingly apply trainable filters and local neighborhood pooling operations on the raw input images, resulting in a hierarchy of increasingly complex features [22, 23].

The first deep autoencoder network was proposed by Hinton and Salakhutdinov in [20]. In contrast to CNNs that apply a series of convolution/pooling/subsampling operations to learn deep feature representations, SAE employs a full connection of units for deep feature learning. SAE contains multiple intermediate layers and millions of trainable parameters that enable it to capture highly nonlinear mapping between input and output, so recently it has been widely applied in some tasks such as image denoising and deconvolution [24, 25], multiple organ detection [26], infant hippocampus segmentation [19], and nuclei regions extraction [27]. Their existing results indicate that the archtecture of SAE is essential for acheiving better performance in a specfic task, which motivates us to investigate the SAE-based feature learning for CVH brain segmentation.

In this paper, we propose a learning-based CVH brain tissues segmentation model that employs unsupervised SAE to automatically learn the deep feature representations and supervised Softmax for classification. Our model is composed of two successive parts: white matter (WM) segmentation module and gray matter (GM)/cerebrospinal fluid (CSF) segmentation module. Specifically, the SAE in two modules takes image patches as input and learns their deep feature representation. These features are then sent to a Softmax classifier for inferring the labels of the center pixels of these patches. To decrease the burden of labeling, only a tiny number of labeled anatomical patches are fed to the network in the training stage. Intuitively, a trained model may be strange to the new patches that contain unknown anatomical structures, but because SAE can learn intrinsic feature representations which are well in eliminating distortion, rotation, and blurring of the input patches, the model can infer the classes of patches that contain unknown anatomical structures. The proposed model was used to segment all 422 CVH brain images at hand. And the segmentation performance of the deep-learned feature was compared with some other representative features (e.g., intensity, PCA, HOG, and AE). Experimental results show that the proposed model achieves higher accuracy in segmenting all three tissues.

The rest of the paper is organized as follows.  Section 2 briefly reviews the acquisition of CVH dataset and the details of the proposed model.  Section 3 reports the experimental results and analyzes the segmentation performance of different SAE architecture. It also compared the performance of different methods and visualizes the three-dimensional reconstruction results. At last, Section 4 concludes the paper.

## 2. Material and Methods

### 2.1. Image Acquisition and Preprocessing

The data utilized in this study are successive cross-sectional images of human brain from the CVH dataset provided by the Third Military Medical University in China. The cadaver is 162 cm in height, 54 kg in weight, and free of organic lesions. Both the donor and her relatives donated their bodies to the Chinese Visible Human program, which follows scientific ethics rules of the Chinese Ethics Department.

**Figure 1:**
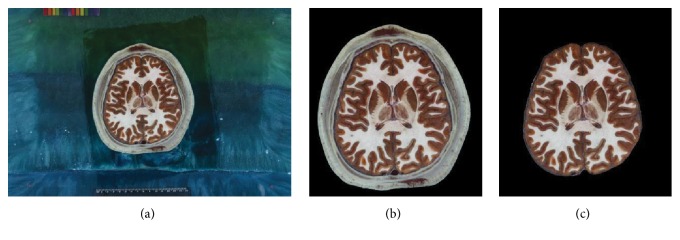
Preprocessing example of cryosection brain image. (a) Original image without any preprocessing (3,072 × 2,048 pixels). (b) Cropped image (1,252 × 1,364 pixels). (c) Skull stripped image.

The images in CVH dataset are taken of the frozen cadaver. A total of 422 cross-sectional images of the head (number 1074 to number 1495) are selected for this study. As shown in Figure 1, the slice is 0.167 mm per pixel, 0.25 mm thick, and photographed at a resolution of 6,291,456 (3,072 × 2,048) pixels with 24-bit color information in tiff format [28]. In order to reduce computational cost and memory usage, these images are transformed into PNG format and cropped to 1,252 × 1,364 pixels. In the preprocessing stage, skull stripping is applied to each image.

### 2.2. Method Overview

**Figure 2:**
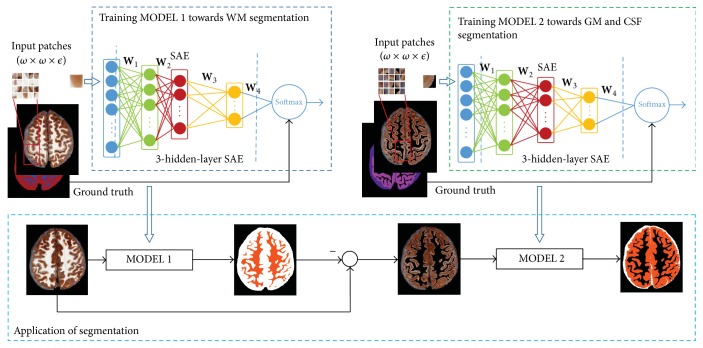
Flowchart of our segmentation model.

In this work, the CVH brain tissue segmentation problem is formulated as a patch classification task and the architecture of our segmentation model is shown in Figure 2. The model takes patches extracted from the B-channel and V-channel of the original images as input, then SAE is used to extract intrinsic feature representation of the input patches, and the following Softmax classifier generates a labels distribution of these patches based on the deep features. This model on segmenting three brain tissues (CSF, GM, and WM) actually contains two submodels: MODEL 1 and MODEL 2. In MODEL 1, GM and CSF are labeled as the same class, and the segmentation is formulated as a three-class classification task: WM, GM & CSF, and background. Through MODEL 1, WM tissue can be extracted from the region of interest. This segmentation result is helpful to fill the areas of WM into background so as to eliminate the influence of WM. So in MODEL 2, the patches from the WM-eliminated image are taken as inputs, and the image is segmented into CSF, GM, and background. This pipeline has the advantages in that more image patches of the objects with fewer labeled data can be taken as it is quite time-consuming to manually label an image. In the following, we will describe the details of the model.

### 2.3. Learning Hierarchical Feature Representation by SAE

#### 2.3.1. Single-Layer AE

A single-layer AE [29] is a kind of unsupervised neural network, whose goal is to minimize the reconstruction error from inputs to outputs via two components: encoder and decoder [19]. In the encoding stage, given an input sample ${\vec{x}}_{n}\in {\mathbb{R}}^{N}$, AE will map it to the hidden activation ${\vec{h}}_{n}\in {\mathbb{R}}^{M}$ by the following mapping:

(1) $$\begin{array}{c} {\vec{h}}_{n}=f\left(\;W_{1}{\vec{x}}_{n}+{\vec{b}}_{1}\;\right), \end{array}$$

where *f*(*z*) = 1/(1 + exp⁡(−*z*)) is a nonlinear activation function; **W**_1_ ∈ *ℝ* ^*M*×*N*^ is the encoder weight matrix; ${\vec{b}}_{1}\in {\mathbb{R}}^{M}$ is the bias vector. Generally, *M* < *N*; then the network is forced to learn a compressed representation of the input vector. This compressed representation can be viewed as features of the input vector. In the decoding stage, AE will reproduce input data from the hidden activation ${\vec{h}}_{n}\in {\mathbb{R}}^{M}$ by

(2) $$\begin{array}{c} {\vec{y}}_{n}=f\left(\;W_{2}{\vec{h}}_{n}+{\vec{b}}_{2}\;\right), \end{array}$$

where **W**_2_ ∈ *ℝ* ^*N*×*M*^ is the decode weight matrix and ${\vec{b}}_{2}\in {\mathbb{R}}^{N}$ is the bias vector.

In our model, during the training stage, we minimize the objective function shown in (3) with respect to the weight matrixes **W**_1_ and **W**_2_ and bias vectors ${\vec{b}}_{1}$ and ${\vec{b}}_{2}$. The objective function includes an average sum-of-square error term to fit the input data and a weight decay term to decrease the magnitude of weight matrices as well as helping prevent over-fitting

(3) JW,b=1m∑n=1m12xn−yn2+λ2∑l=1nl−1 ∑i=1sl ∑j=1sl+1Wjil2,

where **x**_*n*_ denotes the *n*-th sample in the training set; **y**_*n*_ denotes the reconstructed output with input of **x**_*n*_; *λ* denotes weight decay parameter which controls the relative importance of the two terms; *n*_*l*_ denotes the number of layers in the network; *s*_*l*_ denotes the number of units in the *l*th layer; and *m* denotes the number of training samples.

#### 2.3.2. SAE for Hierarchical Feature Learning

**Figure 3:**
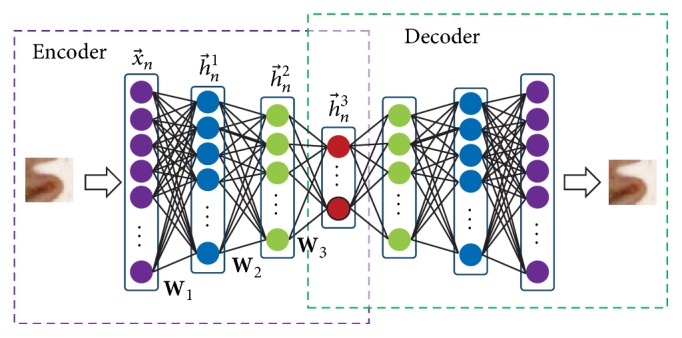
Proposed three-hidden-layer SAE. Note that the number of layers in our model is set via cross-validation.

SAE is a type of neural network consisting of multiple layers of AEs in which the output of each layer is wired to the inputs of the successive layer. In this paper we propose a multi-hidden layer SAE which is shown in Figure 3. It is noted that the number of layers in our model is set via cross-validation. For an input vector ${\vec{x}}_{n}$, the first layer transforms it into a vector ${\vec{h}}_{n}^{1}$ that consists of activations of hidden units, and the second layer takes ${\vec{h}}_{n}^{1}$ as input to produce a new activated vector ${\vec{h}}_{n}^{2}$; then the final activated vector ${\vec{h}}_{n}^{3}$ that is produced by ${\vec{h}}_{n}^{2}$ can be viewed as deep-learned feature representation of the input sample. It is noticed that the model intrinsically handles varying-dimension images through image patches with different sizes. For a specific task, the parameters are usually settled through experiments or experience, and the training and application of SAE will go on those parameters.

In our task, we follow the greedy layer-wise training strategy [18, 20, 30] to obtain better parameters of a SAE. That is, we first train the first single AE on the raw input and then train the second AE on the hidden activation vector acquired by the former AE. The subsequent layers are repeated using the output of each layer as input. Once this phase of training is complete, we stack AEs into SAE and train the entire network by a gradient-based optimization method to refine the parameters.

**Figure 4:**
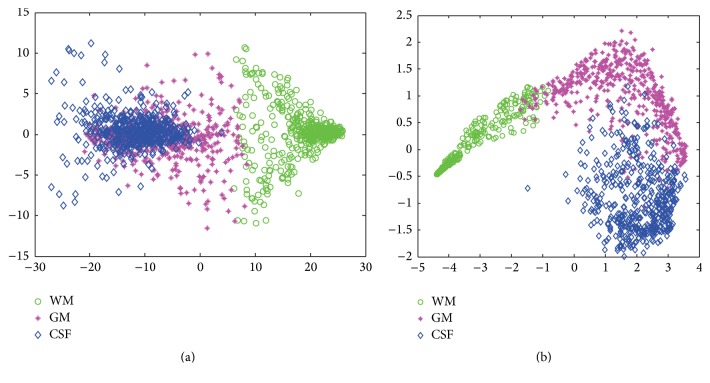
Two-dimensional feature representation for 500 patches of each brain tissue by (a) intensity + PCA and (b) intensity + three-hidden-layer SAE + PCA; here PCA is just for visualization of principle components.

The high-level features learned by SAE are more discriminative compared to hand-crafted feature such as intensity and learning-based feature by single-layer AE. To make an intuitive interpretation, we conducted a dimension reduction experiment to visually examine the distributions of feature vectors from image patches by original intensity and a SAE with three hidden layers, respectively. The experimental result is shown in Figure 4, where the dimensionality of each feature vector is reduced to two by Principal Components Analysis (PCA) for the purpose of visualization. We can see that the features extracted by SAE output a better cluster result than intensity features. It is easier to generate a separation hyperplane for separating different types of samples.

**Figure 5:**
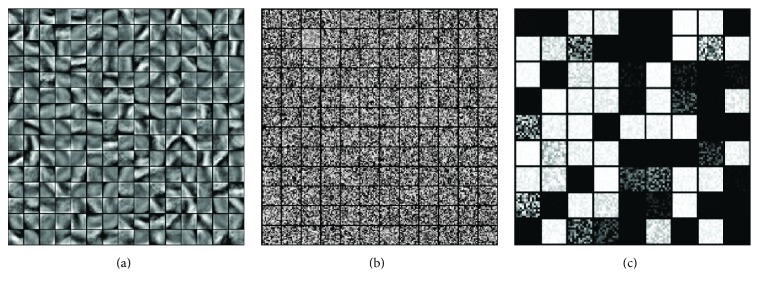
Visualization of learned high-level features of input pixel intensities with three-layer SAE. (a), (b), and (c) Learned feature representation in the first (with 225 units), second (with 144 units), and third (with 81 units) hidden layers, respectively, where the features in the third layer are discriminative for image segmentation task.

To further visualize the discriminative ability between AE and SAE, the features learned by each layer of SAE based on cryosection image are shown in Figure 5. As shown in Figure 5(a), it is seen that AE can only learn primitive oriented edge-like features, just like *K*-means, ICA, or sparse coding do [31]. While SAE can learn higher-level features corresponding to the patterns in the appearance of features in the former layer (as shown in Figure 5(c)), these high-level features are more discriminative for image segmentation task in this paper. Hence, our model employs the SAE instead of both hand-craft features and learning based features by singer-layer AE to extract high-level feature representation for segmenting brain tissues.

#### 2.3.3. SAE Plus Softmax for CVH Brain Tissues Segmentation

For every foreground pixel in the cryosection image, we extract two patches centered at this pixel from its B-channel (in RGB color space) and V-channel (in HSV color space) image, respectively. So *ϵ* in Figure 2 is 2. The two patches are concatenated together as the input features of SAE, and the features learned by SAE then are sent to a supervised Softmax classifier. The parameters of two SAEs in MODEL 1 and MODEL 2 are roughly consistent. The patch size is set via cross-validation, and layer depth is set among {1,2, 3} considering the balance between computation cost and discriminative power. The number of units in each layer of SAE is experimentally set as 400, 200, and 100, respectively. Thus, the final dimensionality of deep-learned feature is 100. The weight decay *λ* of two SAEs in MODEL 1 and MODEL 2 is set to 0.003 and 0.005, respectively, which is tuned on the validation set.

#### 2.3.4. Ground Truth and Training Sets Generation

The objective of the proposed model is to automatically segment the three brain tissues of the whole 422 CVH brain image at hand. It is laborious and time-consuming to manually segment all the brain image for an anatomical expert, so the expert only segmented eight CVH brain images which is saturated with abundant anatomical structures as the ground truth for training sets generation and quantitative evaluation.

The patches used for training and testing are extracted from the eight labeled images. The size of each patch is chosen 17 × 17 to achieve relatively best performance which is validated in Section 3.1, so *ω* in Figure 2 is 17. For MODEL 1, our training sets contain 100,00 WM patches and 90,000 non-WM patches extracted from eight training images. For model 2, the training sets contain 106,000 GM patches and 84,000 CSF patches. These patches are used for SAE and Softmax training in the two models.

## 3. Experimental Results and Discussion

In the experiments, we firstly focus on evaluating the segmentation performance of features learnt by different SAEs based on cryosection image in order to get the best SAE architecture. Secondly, we compare performances of the proposed model based on the deep-learned features and some famous hand-crafted features such as intensity, PCA, and HOG and one learning-based feature by AE. Then, we present typical segmentation examples and make some discussion. Finally, we build the 3D meshes of three tissues based on our segmentation results.

### 3.1. Comparison of Different SAE Architectures

The nonlinear mapping between the input and output of SAE is influenced by its multilayer architecture with various input patch sizes and depths. In order to investigate the impact of different SAE architectures on segmentation accuracy, five different SAE architectures are designed and resort to segmentation task. The detailed parameter configurations are shown in Table 1 and the segmentation performances of WM, GM, and CSF are reported in Table 2.

It can be observed from the results that the predictive accuracies are generally higher for the architectures with input patch sizes of 17 × 17 and 21 × 21. The SAEs with larger patch size tend to have a deeper hierarchical structure and more trainable parameters. These learned parameters are capable of capturing the complex relationship between input and output. We can also observe that the architecture with input patch size of 21 × 21 does not generate substantially higher performance, suggesting that larger patch may introduce more image noise and fused anatomical structures. In order to obtain better segmentation performance, in the following we focus on evaluating the performance of our SAE architecture with input patch size of 17 × 17 and depth of 3.

### 3.2. Comparison of Performances Based on Different Features

In order to provide a comprehensive evaluation of the proposed method and illustrate the effect of high-level features in contrast to low-level features, three representative hand-crafted features such as intensity, PCA [32, 33], and HOG [34] and one learning-based features by AE are used for comparison. These features follow the same segmenting procedures as the deep-learned features. All the segmentation performances are reported in Table 3 using Dice ratio. It can be observed that our model with the features extracted by SAE outperforms other well-known features for segmenting all three types of brain tissue. Specifically, our model yields the best average Dice ratio of 90.69 ± 2.14% (CSF), 91.24 ± 2.01% (GM), and 96.12 ± 1.23% (WM) in a leave-one-out evaluation manner. These results have illustrated the strong discriminative power of the deep-learned features in brain tissues segmentation task.

**Figure 6:**
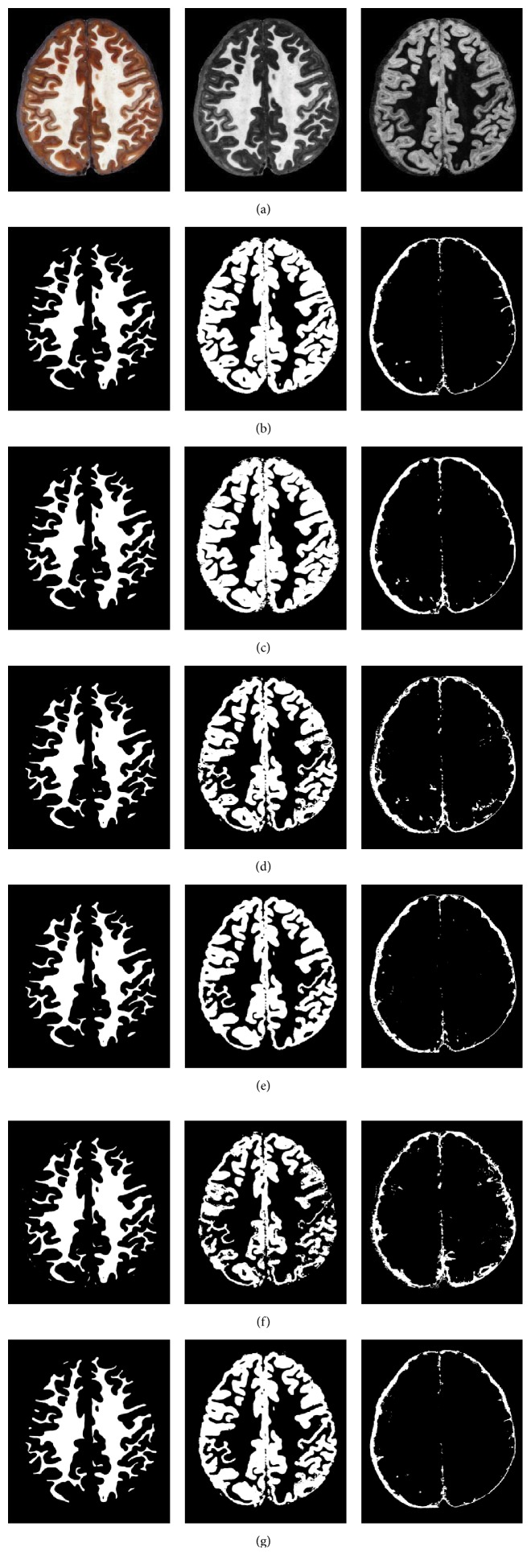
Comparison of the segmentation results with the manually generated segmentation on a cryosection image in CVH dataset. (a) shows the original cryosection and its B-channel (in RGB color space) and V-channel (in HSV color space) image. (b) shows the manual results (CSF, GM, and WM). (c)–(g) show the results by the features of our SAE deep learning, Intensity, PCA, HOG, and AE, respectively.

To further demonstrate the advantages of our proposed model, we visually examine the segmentation results on one cryosection image which is shown in Figure 6. (a) shows the original RGB cryosection image and its B- and V-channel images. The ground truth that segmented by experts is shown in (b). (c)–(g) present segmentation results of four methods based on deep-learned features, intensity features, PCA features, HOG features, and AE feautres, respectively. We can see that the segmentation results of the proposed model are quite close to the ground truth. In contrast, other results either generate much oversegmentation or fail to segment tiny anatomical structures accurately. Specifically, WM is only adjacent to the GM and can be easily distinguished from its surroundings, so the WM segmentation DRs are approximate to each other. And the visible results of WM are similar in appearance except the fact that HOG-based method mistakenly introduced a small fraction of CSF into the results. It has some difficulty on GM and CSF segmentation due to the complex anatomical structures and low contrast. HOG and intensity based methods introduce more surrounding non-GM or non-CSF tissue into the ROI of GM or CSF, respectively; thus they produce more defects and fuzzy boundaries for different tissues. In contrast, the GM and CSF tissues generated by our method can be clearly identified with a certain ROI and distinct contours.

**Figure 7:**
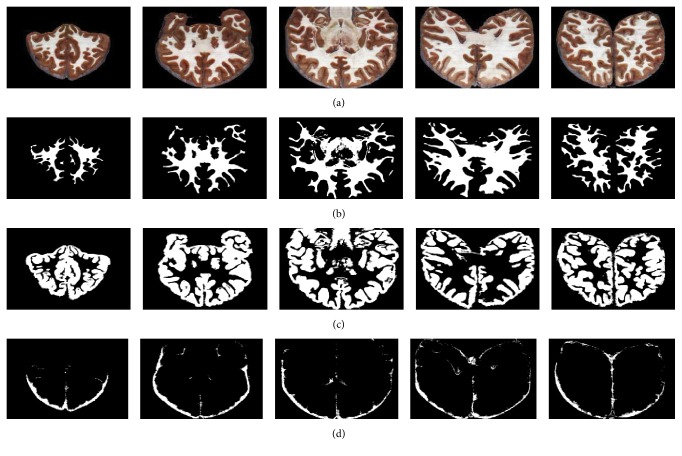
Coronal section images (a) and their corresponding SAE segmentation results of WM, GM, and CSF ((b), (c), and (d)).

**Figure 8:**
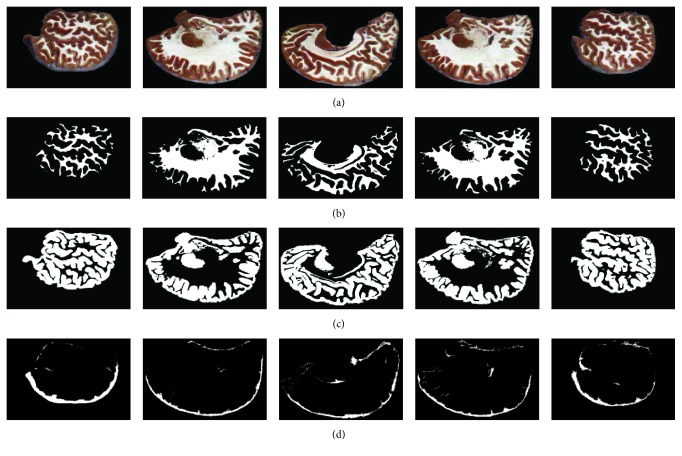
Sagittal section images (a) and their corresponding SAE segmentation results of WM, GM, and CSF ((b), (c), and (d)).

We then applied our proposed model to segment all 422 brain cryosection images. Typical images in coronal and sagittal viewpoints and their corresponding segmentation results (WM, GM, and CSF) are shown in Figures 7 and 8, respectively. It is remarkably seen that the results of WM and GM change continuously and their morphological distributions are shown clearly; most tiny anatomical structures are well reserved. It is also noticed that the results of CSF seem incomplete and not distributed uniformly. This fact is determined by the characteristics of the cryosection images. These images have such high spatial resolution (0.167 mm per pixel and 0.25 mm per slice) that it can express fine structures of tissues. But since these images were collected from a cadaver, the CSF in the brain no longer flowed in vivo. Due to the effect of gravity, the CSF will gather to some places, rather than uniformly distributing around the surface of brain as in live status. These factors cause the discontinuous distribution of the CSF segmentation results.

### 3.3. Three-Dimensional Reconstruction Results

**Figure 9:**
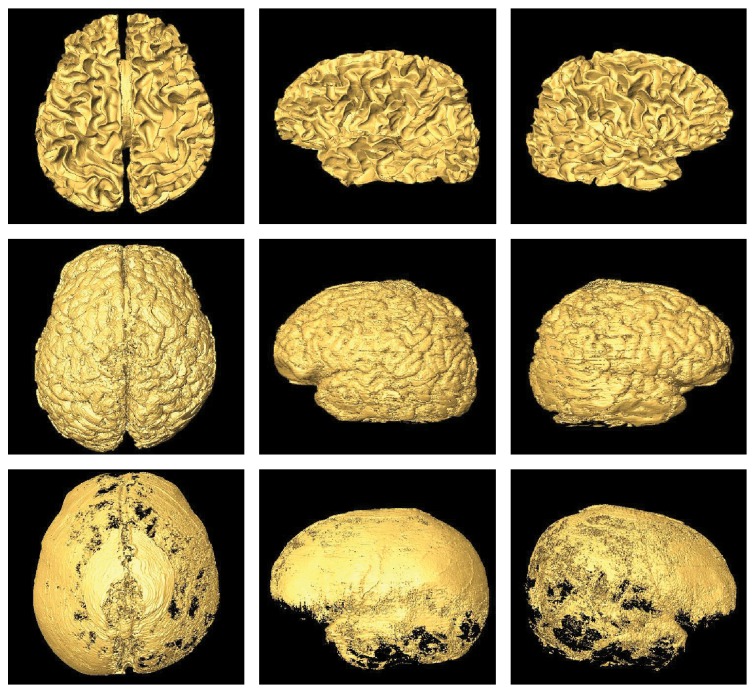
Three-dimensional surface-rendering reconstruction results of WM, GM, and CSF based on our segmented images.

The CVH cryosection slices are 0.167 mm per pixel and 0.25 mm thick, and such high resolution is very helpful for displaying subtle anatomical structures of brain tissues. For a more in-depth understanding of these tissues, the segmented white matter, gray matter, and cerebrospinal fluid images are reconstructed using marching cubes algorithm to produce 3D surface mesh. The reconstruction results in different views are shown in Figure 9. From the surface-rendering reconstruction results, it is seen that the surface of WM is smooth and its sulci and fissures are clearly displayed. The distribution of GM shape is also noticed, but the surface of GM reconstruction results does not look very smooth. The reason for this lies in the fact that the GM and CSF are mixed together because of the ice crystals in the frozen brain slices, so the segmentation of GM is influenced by its surrounding CSF. In spite of it, the sulci and cerebral cisterns are also easy to be recognized in 3D reconstructed WM and GM.

Benefitting from the increasing development of the 3D reconstruction technology, 3D MRI and PET have now been used in clinic and researches. But because of the resolution limitation and complexity of brain structures of the 2D radiological images (such as CT and MRI), the 3D reconstruction results are usually unsatisfactory and are hard for the guidance of clinic operation. For the work in our paper, we focus on the segmentation and reconstruction of three kinds of CVH brain tissues. The CVH brain images have high spatial resolution of 0.167 mm per pixel and 0.25 mm per slice, after segmentation by the proposed method; the high-quality and high-accuracy 2D brain tissues can get well 3D reconstruction results. Some potential applications of the 3D reconstruction result include the following:

1. The 3D results can be viewed in any orientation besides the common coronal, sagittal, and transverse orientations. These 3D models (especially for WM and GM) can help obtain the anatomical knowledge of 3D structures and their adjacent relationship in space. In addition, we can identify the structures by comparing radiological image with the anatomical image.
2. The 3D result in our work is applicable for teaching sectional anatomy since there is rarely direct-viewing model that can make the understanding of anatomical structures easier. The varying viewpoints of the 3D model are helpful for observing the tiny structures in specific positions of human brain, and medical students only need to move their mouse to control the perspective of displaying.

## 4. Conclusion

We have presented a supervised learning-based CVH brain tissue segmentation method using the deep-learned features with multilayer SAE neural network. The major difference between our proposed feature extraction method and conventional hand-crafted features such as intensity, PCA, and Histogram of Gradient (HOG) is that it can dynamically learn the most informative features adapted to the input dataset at hand. The discriminative ability of our proposed model is evaluated and compared with other types of image features. Experimental results validate the effectiveness of our proposed method and show that it significantly outperforms the methods based on typical hand-crafted features. In addition, the high-resolution 3D tissue surface meshes are reconstructed based on the segmentation results by our method with the resolution of 0.167 mm per pixel and 0.25 mm per slice, much more tiny than the 3 T (even 7 T) MRI brain images. Furthermore, since the procedure of features extraction by our method is independent of the CVH dataset, our method can be easily extended to segment other medical images such as cell images and skin cancer images. CVH dataset contains serial transverse section images of the whole human body, which is large in volume. Pure manual or semiautomatic segmentation of those images is quite time-consuming, so a large proportion of data still remain to be exploited. Though the work in our paper only had segmented the WM, GM, and CSF of the brain tissue, it actually provides a reference for automatically or semiautomatically processing such real-color and high-resolution images.

Recent studies show that neural network can yield more promising performance on image recognition task with deeper hidden layers [35, 36]; we will explore parallel SAE with more hidden layers as well as more training data in the future. In addition, the number of neural units in each hidden layer may affect the segmentation performance in a certain degree. We will further investigate the influence of hidden neural units to segmentation performance. Furthermore, the model uses a classical Softmax classifier to predict labels of the input patches, and we will consider the influence of different classifiers in the future research.

## Figures and Tables

**Table 1:** Details of the SAE architectures with different input patch size and layer depth in this study.

Patch size		Layer 1	Layer 2	Layer 3	Layer 4
5 × 5	Layer type	AE	Softmax	—	—
	Input size	50	25	—	—
	Hidden size	25	—	—	—

9 × 9	Layer type	AE	AE	Softmax	—
	Input size	162	81	36	—
	Hidden size	81	36	—	—

13 × 13	Layer type	AE	AE	Softmax	—
	Input size	338	150	64	—
	Hidden size	150	64	—	—

17 × 17	Layer type	AE	AE	AE	Softmax
	Input size	578	400	200	100
	Hidden size	400	200	100	—

21 × 21	Layer type	AE	AE	AE	Softmax
	Input size	882	400	200	100
	Hidden size	400	200	100	—

**Table 2:** Mean and standard deviation of Dice ratio (in %) for measuring the performance of the three tissue types with five different architectures trained by using different patch sizes of 5 × 5, 9 × 9, 13 × 13, 17 × 17, and 21 × 21, respectively. The experiments were conducted in a leave-one-out manner and eight test results were collected for each tissue.

Patch size	WM	GM	CSF
5 × 5	92.41 ± 2.85	90.36 ± 3.24	88.41 ± 3.01
9 × 9	93.95 ± 2.48	90.45 ± 2.13	89.54 ± 2.57
13 × 13	94.25 ± 2.01	91.74 ± 2.51	90.14 ± 2.84
17 × 17	96.12 ± 1.63	92.24 ± 2.11	90.69 ± 2.14
21 × 21	96.64 ± 1.85	91.12 ± 2.36	90.45 ± 2.04

**Table 3:** Mean and standard deviation of Dice ratio (in %) for the segmentations obtained by five feature representation methods.

Method	CSF	GM	WM
Intensity	88.76 ± 2.41	89.46 ± 2.12	95.49 ± 1.96
PCA	89.31 ± 1.89	90.87 ± 2.34	95.20 ± 2.01
HOG	86.77 ± 2.67	87.21 ± 2.86	94.92 ± 1.89
AE	88.32 ± 2.37	90.26 ± 2.36	95.60 ± 1.83
Proposed SAE	90.69 ± 2.14	91.24 ± 2.01	96.12 ± 1.23
